# The CD70-CD27 axis in oncology: the new kids on the block

**DOI:** 10.1186/s13046-021-02215-y

**Published:** 2022-01-06

**Authors:** Tal Flieswasser, Astrid Van den Eynde, Jonas Van Audenaerde, Jorrit De Waele, Filip Lardon, Carsten Riether, Hans de Haard, Evelien Smits, Patrick Pauwels, Julie Jacobs

**Affiliations:** 1 Center for Oncological Research (CORE), Integrated Personalized and Precision Oncology Network (IPPON), Wilrijk, Belgium; 2 Department of Pathology, Antwerp University Hospital, Edegem, Belgium; 3 Department of Medical Oncology, Inselspital, Bern University Hospital, University of Bern, Bern, Switzerland; 4 Argenx, Zwijnaarde, Ghent, Belgium; 5 Center for Cell Therapy and Regenerative Medicine, Antwerp University Hospital, Edegem, Belgium

**Keywords:** CD70, CD27, Hematopoiesis, Oncology, Hematological malignancies, Solid tumors, Combination therapies

## Abstract

The immune checkpoint molecule CD70 and its receptor CD27 are aberrantly expressed in many hematological and solid malignancies. Dysregulation of the CD70-CD27 axis within the tumor and its microenvironment is associated with tumor progression and immunosuppression. This is in contrast to physiological conditions, where tightly controlled expression of CD70 and CD27 plays a role in co-stimulation in immune responses. In hematological malignancies, cancer cells co-express CD70 and CD27 promoting stemness, proliferation and survival of malignancy. In solid tumors, only expression of CD70 is present on the tumor cells which can facilitate immune evasion through CD27 expression in the tumor microenvironment. The discovery of these tumor promoting and immunosuppressive effects of the CD70-CD27 axis has unfolded a novel target in the field of oncology, CD70.

In this review, we thoroughly discuss current insights into expression patterns and the role of the CD70-CD27 axis in hematological and solid malignancies, its effect on the tumor microenvironment and (pre)clinical therapeutic strategies.

## Background

In the last decade, the emergence of immunotherapy has revolutionized the treatment of hematological and solid cancers by relying on cancer destruction through activation of the host’s immune system [1, 2]. In solid tumors, immune checkpoint inhibitors, such as anti-CTLA-4, anti-PD-1 and anti-PDL1 have demonstrated the therapeutic potential of immunotherapy, resulting in Food and Drug Administration (FDA)/European Medicines Agency (EMA) approvals, and have become the standard of care in over 50 types of cancer as single agents or in combination strategies [3]. While clinical effectiveness of immune checkpoint inhibition is quite limited in hematological tumors (except Hodgkin lymphoma), chimeric antigen-receptor (CAR) Tcells are reshaping the field of hematological malignancies and have led to FDA/EMA approvals since 2017, especially against the B cell antigen CD19 [4]. However, the sobering reality is that generally speaking only a minority of patients benefit from long-term remission [5, 6].

New immunotherapeutic targets could lead to more effective treatment alternatives as single agents or in rationally designed combination strategies. In this regard, the CD70-CD27 axis, belonging to the tumor necrosis factor (TNF) superfamily, has become an attractive target to exploit in oncology [7]. In physiology, the receptor, CD27, is generally found on naive T and memory B and T cell populations and subsets of natural killer (NK) cells. On the other hand, CD70 is only transiently expressed on antigen-activated B and T cells, NK cells and mature dendritic cells. Upon activation of CD27 by CD70, the extracellular domain of CD27 is cleaved off and found as a soluble fragment (called sCD27) in body fluids [8]. In oncology, CD70 is aberrantly expressed on malignant cells without (solid tumors) or with CD27 co-expression (hematological malignancies), facilitating immune evasion through the tumor microenvironment (TME) and tumor progression [7, 8]. In addition, high levels of sCD27 have been found in serum samples of patients with hematological and solid malignancies, suggesting the involvement of the CD70-CD27 axis [9–14]. This review discusses the role of the CD70-CD27 axis during normal hematopoiesis, the biological effect of its dysregulation in hematological and solid malignancies and the promising role of CD70 as target in the clinic.

### The CD70-CD27 axis in hematopoiesis

In hematopoiesis, the hematopoietic stem cells (HSCs) balance self-renewal capacity with multilineage potential. In this way, durable cell levels can be maintained while giving rise to progenitor, precursor and eventually fully differentiated blood cells of the myeloid and lymphoid lineage [15]. A role of the CD70-CD27 axis in the early development stages could only be demonstrated in mice. Multiple studies observed CD27 expression on murine HSCs and progenitor/precursor cells [16–19]. Moreover, Nolte et al. [20] showed that the interaction of CD27 positive (CD27^+^) progenitor cells with CD70 inhibited leukocyte differentiation [20]. Therefore, it has been suggested that the CD70-CD27 axis mediates a negative feedback system, enabling the activated immune system to modulate hematopoiesis [20]. With regard to human hematopoiesis, there is no hard evidence of CD27 and/or CD70 expression on human HSCs and progenitor cells [12, 21], suggesting that the early development stages are independent of the CD70-CD27 axis*.* By way of contrast, expression of both CD70 and CD27 is found on their malignant counterparts (further discussed in the next section).

Knowledge on expression patterns of CD70 and/or CD27 on more differentiated cells indicate a role for the CD70-CD27 axis during downstream hematopoiesis, however the underlying mechanisms are not yet uncovered. Some studies report the expression of CD27 after commitment to the lymphoid lineage on human B cell progenitor/precursor populations [22–24]. While research on circulating immature and naive B cells showed no CD27 expression, CD70 was found to be transiently upregulated on activated B cells upon antigen encounter. Interestingly, studies investigating B cell differentiation found that CD70-CD27 interactions are important in formation of memory and plasma B cells. Accordingly, within the germinal centers of secondary lymphoid tissue, expression of CD27 is moderate on B cells, upregulated on plasma cells and maintained on a large subset of memory B cells [25–28]. On the other hand, CD70 expression has only been reported on a small subset of germinal center B cells [27, 29].

In human thymocyte development, CD27 expression could only be detected on the latest stage [30, 31]. Moreover, a few studies showed that human thymic stromal cells that provide essential signals for T cell development and clonal selection express low levels of CD70 [29, 32]. The function of the CD70-CD27 axis in human thymic development still needs to be uncovered. In mice it is shown that this interaction is not essential for the generation of conventional CD4^+^ and CD8^+^ αß T cell populations [33], while it is important in the functional differentiation of the low abundant γδ T cell subsets [34]. Opposite to naive B cells, multiple studies reported CD27 expression on almost all naive T cells and subsequent activation resulted in CD70 upregulation which diminished again after gaining full effector functions [35, 36]. As seen for B cells, CD27 expression is present on regulatory T cells (Tregs) [37] and on memory T cells [33, 38].

Besides B and T cells, CD70 and CD27 expression is also found to be strictly regulated on human NK cells. Here, it could be demonstrated that CD27 is upregulated during the latest stage of development and is downregulated on the majority after gaining effector functions. Similarly to previous results on B and T cells, CD70 is only transiently upregulated upon NK cell activation [39, 40].

Although more research is necessary to unravel the molecular mechanisms during early cell development, CD70 and CD27 on mature immune cells operate as costimulatory molecules and their interaction contributes to immune regulation via different signaling pathways. It is seen that CD27 signaling activates the NF-κB and c-Jun kinase pathways via bound T RAF2/5, leading to cell proliferation, survival and differentiation. Additionally, triggering of CD27 can result in apoptosis as well via the receptor-associated death domain-containing adaptor protein Siva [8]. Finally, it was shown by different studies that CD70 can exert reverse signaling through induction of PI3K/Akt and MEK signaling pathways regulating cell expansion, differentiation and effector functions [41–44].

**Fig. 1:**
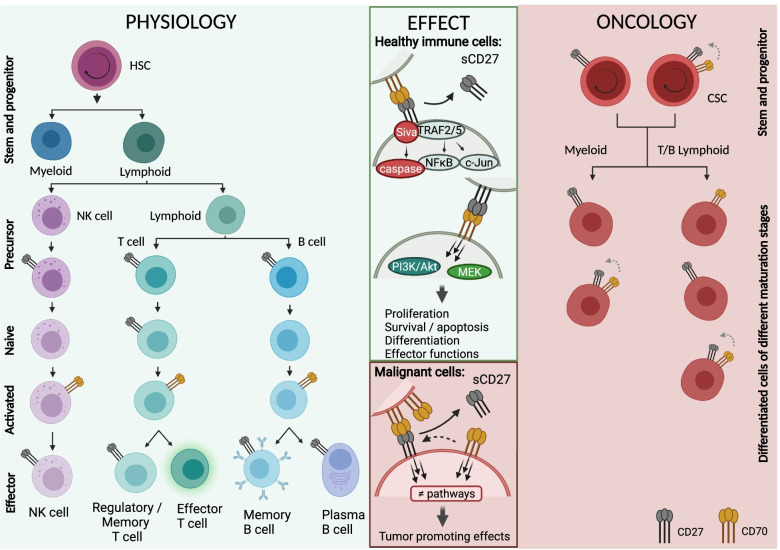
The CD70-CD27 axis in hematopoiesis and oncology. During normal hematopoietic development, expression of CD70 is tightly regulated and plays a role in priming, survival and differentiation for a subset of immune cells through NF-κB and c-Jun pathway activation via TRAF 2/5 leading. Alternatively, apoptosis can be induced by activating the caspase pathway via Siva. Reverse CD70 signaling can activate PI3K/Akt and MEK signaling pathways leading to regulation of cell expansion, differentiation and effector functions. In oncology CD70 and CD27 are frequently co-expressed on malignant cells which can deliver proliferation signals and a growth advantage to the malignant cells. Apart from improved growth, dysregulation of the axis can provide other tumor promoting effects via different signaling pathways. Figure created with BioRender.com. Abbreviations; CSC, cancer stem cell; HSC, hematopoietic stem cell; MEK, mitogen-activated protein kinase kinase; NFκB, nuclear factor kappa B; NK cell, natural killer cell; PI3K, phosphoinositide 3-kinase; sCD27, soluble CD27; TRAF 2/5, TNF receptor associated factor 2/5

Altogether these data show that the CD70-CD27 axis is tightly regulated during hematopoietic cell development showing either CD70 or CD27 expression but generally never co-expression of both markers. In addition, interaction of these costimulatory molecules orchestrates important signaling pathways on mature B/T cells and NK cells shaping immune responses. Given the biological functions of the CD70-CD27 axis, it is not surprising that acquisition or overactivation of the axis by abnormal expression patterns can contribute to malignancy. In the next paragraph, the aberrant expression patterns and associated signaling that has been found for a considerable number of hematologic and solid tumors will be discussed. An overview of expression patterns and signaling in physiology and oncology is depicted in Fig. 1.

### The CD70-CD27 axis in oncology

#### Expression on hematologic malignancies

##### Leukemia

Leukemic stem cells (LSCs) can be seen as the malignant counterparts of HSCs as they account for initiation and maintenance of the disease, and share self-renewal capacity and multipotency to give rise to more differentiated malignant cells [15]. Although absent from normal HSCs, constitutive expression of CD27 is detected on LSCs of acute myeloid leukemia (AML) and chronic myeloid leukemia (CML) patients [12, 21, 45]. In addition, CD70 is co-expressed on LSCs from AML patients [12]. Co-expression of CD70 and CD27 is also present on AML [12] and B cell acute lymphoblastic leukemia (B-ALL) blasts [10, 22, 46, 47], on malignant B cells of chronic lymphocytic leukemia (B-CLL) and subtypes including hairy cell leukemia and its prolymphocytic variant [10, 48–51]. Finally, high levels of sCD27 were detected in serum samples from AML patients and were associated with inferior prognosis [12].

##### B cell lymphoma and multiple myeloma

The presence and relevance of LSCs in CML and AML is generally accepted, but whether other hematologic malignancies arise from similar populations is still unclear [52]. Nonetheless, CD27 is identified on a rare B cell population that may be responsible for the generation and maintenance of the characteristic Reed-Sternberg cells of Hodgkin lymphoma and on a population suggested to be multiple myeloma stem cells [53–55].

Co-expression of CD70 and CD27 can be found on the surface of malignant B cells in non-Hodgkin lymphomas (NHL) such as diffuse large B cell lymphoma (DLBCL) [7, 51, 56], follicular lymphoma [56, 57], follicle center lymphoma [51], mantle cell lymphoma [7, 50, 51, 58, 59], Burkitt lymphoma [51], and Waldenström macroglobulinemia [60]. Of interest, high levels of CD70 were related to an unfavorable outcome with a shorter overall survival in different types of DLBCL [61]. In addition, as seen for AML, sCD27 has been identified as a prognostic factor for different NHL subtypes [11, 14]. In Hodgkin lymphoma, a different expression pattern was observed whereby Reed-Sternberg cells show high CD70 levels but lack the expression of CD27 [54, 62, 63].

Expression of CD70 and CD27 is also detected on malignant plasma cells in multiple myeloma, where CD27 decreases with increasing progression of the disease and its absence might even be a prognostic factor for high-risk disease [64, 65]. The related plasma cell leukemia by way of contrast shows robust CD27 expression [66].

##### T cell lymphoma

Constitutive CD27 and CD70 expression has also been detected on T cell neoplasia such as anaplastic large cell lymphoma [7, 67], peripheral T cell lymphoma [7], cutaneous T cell lymphoma [68–72], adult T cell leukemia/lymphoma [73–75] and extranodal NK/T cell lymphoma [7, 76, 77]. On the contrary, only CD70 expression is observed on chronic active Epstein-Barr virus associated T cell lymphoma (a rare complication of latent Epstein-Barr virus infection) [78] and T cell acute lymphoblastic leukemia [79].

#### Signaling in hematologic malignancies

The strict expression patterns of CD70 and CD27 during normal physiology allow for regulation of immune responses via immune cell proliferation, differentiation, survival and death depending on the situation and timing. Hematological malignancies show aberrant expression of both molecules leading to dysregulated signaling of the axis, thereby providing the malignant cells with tumor-promoting capacities.

In leukemia, it is seen that the CD27 signaling on AML and CML LSCs leads to induction of the Wnt pathway, an important pathway for self-renewal [12, 21, 45]. Schürch et al. [21] revealed that the aberrant activation of the Wnt pathway occurs through ß-catenin activation, a central component of the pathway in the cytoplasm [21]. This impaired Wnt activation resulted in increased proliferation of LSCs [12, 21] and drug resistance [45], both promoting leukemic progression.

Furthermore, the study of Riether et al. [12] observed that CD70/CD27 signaling on immature AML blasts induced stem cell gene signatures through the canonical Wnt pathway (as discussed above), JAK/STAT pathway, Hedgehog pathway and transforming growth factor beta (TGF-ß) signaling. Functional analysis showed that the CD70/CD27 interaction was also responsible for symmetric cell divisions, promoting the immature blast state and preventing myeloid differentiation [12]. The involvement of the CD70-CD27 axis in blastoid formation was also seen in a rare case of low-grade B cell lymphoma, where upregulation of CD70 and CD27 coincided with a highly increased gene expression of galactin 1 and TGF-ß receptor III, suggesting this could be implicated in the MEK and TGF-ß signaling pathway, respectively [80]. Related to this case, CD70 was higher expressed on the blastoid variant of mantle cell lymphoma, a clinically more aggressive form defined by a higher mitotic rate compared to the common mantle cell lymphoma [59].

Additionally, there is increasing evidence that the CD70-CD27 signaling promotes tumor cell proliferation. In leukemia, blocking of the CD70-CD27 interaction in CML [21], AML [12], B-ALL [22] and some cases of B-CLL [51] reduced proliferation of the malignant cells. This effect on proliferation was also seen in B cell lymphoma where CD27 crosslinking increased proliferation of cell lines through augmented protein kinase C activation [81]. Apart from CD27 signaling, CD70 reverse signaling was also reported to increase proliferation in a low-grade B cell lymphoma. Here, CD70 was hinted to have a responsive state (signaling) during remission phases and a non-responsive state during attack [80]. Interestingly, binding of sCD27 to CD70 was shown to induce proliferation on extranodal NK/T cell lymphomas [76].

Finally, the CD70-CD27 axis can affect malignant cell survival. As discussed above, ligation of CD70 with CD27 on CML LSCs mediated drug resistance by compensatory Wnt pathway activation [45]. This increased survival was also observed in plasma cell leukemia where triggering of CD27 with CD70 rescued plasma cells from drug-induced apoptosis via regulation of p38 and ERK 1/2 MAP kinases of the MEK pathway and the downstream transcription factor AP-1 [66]. In addition, modulation of both anti- and pro-apoptotic proteins, including the Bcl-2 family, has been described to be dependent of the presence of CD70/CD27 on malignant lymphoma cells. However, the exact role of CD70/CD27 in the regulation of survival needs further elucidation [59, 65, 80].

**Fig. 2:**
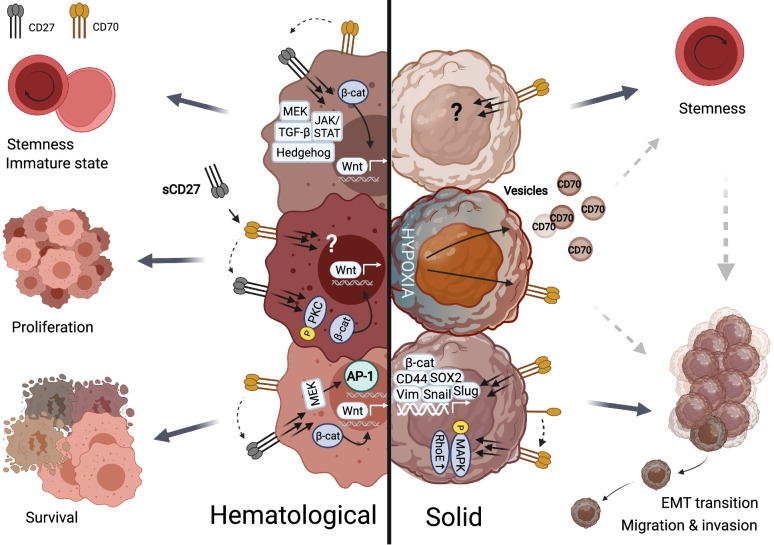
Signaling pathways of the CD70-CD27 axis in hematologic and solid cancers. In hematological malignancies, CD70-CD27 signaling activates the canonical Wnt, JAK/STAT, Hedgehog and TGF-ß pathways which can induce stemness/immature state. In addition, crosslinking of CD27 in a protein kinase C or ß-catenin-dependent manner and reverse signaling of CD70 were shown to induce proliferation of the malignant cells. CD70-CD27 signaling can promote survival by regulating kinases of the MEK pathway and transcription factor AP-1 and by the Wnt pathway through activation of ß-catenin. In solid tumors, CD70 signaling is associated with cancer stem cells and EMT transition via the induction of EMT-related gene expression (SOX2, CD44, Vimentin, Snail, Slug and ß-catenin) and via MAPK activation and RhoE overexpression. Furthermore, hypoxia is identified as a regulator of CD70 expression and is an important factor to promote stemness, migration and invasion of the tumor. Figure created with BioRender.com. Abbreviations; AP-1, activator protein 1; ß-cat, ß-catenin; EMT, epithelial to mesenchymal transition; JAK/STAT, Janus kinase-signal transducer and activator of transcription; MAPK, mitogen-activated protein kinase; MEK, mitogen-activated protein kinase kinase; PKC, protein kinase C; RhoE, ras homologous E; sCD27, soluble CD27; SOX2, sex determining region Y-box2; TGF-ß, transforming growth factor beta; Vim, Vimentin

Hence, these different studies report the CD70-CD27 signaling axis as a driver of malignancy in hematologic malignancies supporting stemness, proliferation and survival. An overview of the signaling pathways involved in both hematologic and solid cancers is depicted in Fig. 2.

#### Expression on solid tumors

Over the past decades, the importance of CD70 on solid tumors has become clear whereby aberrant expression of CD70 on tumor cells has been reported on numerous types of solid tumors with varying expression levels [8]. As such, CD70 expression has been reported on both primary and metastatic tumor resections in renal cell carcinoma, nasopharyngeal carcinoma, glioblastoma, melanoma, lung carcinoma, cervix carcinoma, breast carcinoma, ovarian carcinoma and mesothelioma and was associated with decreased survival [9, 82–86]. Interestingly, CD70 expression was found to be even higher in metastatic specimens of lung carcinoma, pancreatic carcinoma and osteosarcoma, suggesting the importance of CD70 in progression of the disease [7]. Consequently, therapies targeting CD70 hold great potential to combat both early and advanced stages of cancer. On the contrary, CD70 seems (almost) absent on the tumor cells in Kaposi sarcoma, prostate carcinoma, Langerhans cell histiocytosis and colorectal carcinoma [8, 87]. Although CD70 is prevalent in numerous cancers, CD70 expression patterns may vary among these different tumor types in spatial distribution, intensity of expression and percentage of positive cells.

In contrast to hematological malignancies, expression of CD27 on the tumor cells has never been reported in solid tumors. Nonetheless, CD70-CD27 signaling can occur through the presence of CD27 in the TME facilitating immune evasion and tumor progression by distinct mechanisms [88–91].

#### Signaling in solid tumors

The role of CD70 reverse signaling in tumor progression has already been described in hematological malignancies but is rather undefined in solid tumors. It was reported in melanoma that CD70 expression regulates invasion and metastasis intrinsically, rather than through the TME. Here the study of Pich et al. [91] showed that signaling of CD70 was dependent on its oligomerization. While the monomeric form of CD70 limited the metastatic and migratory ability of melanoma cells, the trimeric form of CD70 enhanced cell migration and invasiveness [91].

Apart from hematological tumors, cancer stem cells (CSCs) have also been identified in solid tumor types where they are recognized as important players in metastasis, recurrence, heterogeneity and drug resistance [92]. Although for many cancers the presence of CSCs is currently under debate, its existence in glioblastoma, breast cancer and colon cancer is now generally accepted [93]. The involvement of CD70 in the generation and/or maintenance of a CSC phenotype has been described in breast cancer, glioblastoma, melanoma, pancreatic carcinoma and non-small cell lung cancer (NSCLC) and thus could serve as a potential marker of stem cells [12, 94]. In breast cancer, these CD70 expressing CSCs displayed a mesenchymal phenotype, self-renewal potential and enhanced metastasis to the lung compared to the CD70^−^ CSCs population that exhibited an epithelial phenotype [94]. CD70 has also been associated with tumor epithelial to mesenchymal transition (EMT), a process by which epithelial cells gain migratory and invasive characteristics in glioblastoma, melanoma, pancreatic carcinoma and NSCLC [91, 95, 96]. Upon silencing of CD70 in glioblastoma cell lines, expression levels of EMT-associated genes, SOX2 and CD44, decreased, resulting in inhibition of tumor growth and migration [95]. In addition, it was shown that β-catenin was significantly reduced after siRNA-mediated knock-down of CD70 expression in human pancreatic cell lines, as well as other EMT-related genes, such as Vimentin, Snail and Slug [97]. In melanoma, CD70 expression led to MAPK activation and RhoE overexpression, thereby promoting tumor migration [91].

Hypoxia is an important factor of EMT, stem-cell maintenance, invasion, metastasis, angiogenesis and resistance to therapy in solid tumors [98]. In renal cell carcinoma, CD70 induction was reported under hypoxic conditions [99, 100]. In support of this notion, a strong constitutive CD70 expression has been described within hypoxic regions of a murine model for NSCLC [101]. Furthermore, it has been reported that hypoxia can increase the number of small protein-carrying packets called vesicles, which regulate inter-cellular communication, leading to changes in the biological activity. Interestingly, a recent study has shown that in the context of lung cancer, hypoxic conditions stimulated the synthesis of tumor vesicle proteins such as CD70, thereby possibly supporting immune suppression [98].

Altogether, these findings underline an important role of CD70 signaling in acquiring aggressive traits in solid tumor types (an overview is shown in Fig. 2).

### The CD70-CD27 axis and the tumor microenvironment

Along with a pivotal role of the CD70-CD27 axis on malignant cells, dysregulation of these costimulatory molecules can also cooperate to escape anti-tumor immune surveillance in hematological and solid tumor types by distinct mechanisms in the TME.

In glioma and renal cell carcinoma, the CD70-CD27 axis has shown to mediate apoptosis of lymphocytes [89, 102, 103]. Siva is thought to induce apoptosis of T cells by initiating caspase activation after binding of CD70 to CD27 [104, 105]. Next to apoptosis mediated through CD27 signaling, TGF-β is also known to induce apoptosis in a variety of cell types [90]. The study of Yang et al. [90] showed that treatment with TGF-β induced apoptosis of exhausted CD70 positive (CD70^+^) T cells at a significantly higher rate than CD70^−^ T cells in NHL by acquiring more pro-apoptotic markers, such as caspase-3 [90].

T cell exhaustion is another mechanism by which CD70-CD27 signaling can reduce immune surveillance. In renal cell carcinoma, tumor infiltrating lymphocytes were found to have an exhausted phenotype, which was driven by CD70 expression [106]. Furthermore, TGF-β induced upregulation of CD70 expression via Smad3 and IL-2/STAT5 signaling, resulting in exhaustion of effector memory T cells in B cell NHL. Both TGF-β-induced, as well as pre-existing intratumoral CD70^+^ T cells, were found to be functionally exhausted with lower proliferation, signaling transduction, cytokine production and higher expression levels of PD-1 and TIM-3 after treatment with TGF-β compared to CD70^−^ T cells [90]. Whether the interaction between CD70 and CD27 plays a role in TGF-β-induced T cell exhaustion is still not clear. Yet, Yang et al. [90] found TGF-β mediated downregulation of CD27 on CD70^+^ T cells due to shedding of CD27. This loss of CD27 on the cell surface was reversed after blocking of CD70 and led to restored cell function and viability, indicating a potential role of the CD70/CD27 interaction in TGF-β-mediated T cell exhaustion [90].

Thirdly, the CD70-CD27 pathway can mediate immune escape through its effects on Tregs. CD27 signaling resulted in a direct decrease of Treg apoptosis in murine solid tumor models and an indirect increase of Tregs in vitro, mediated via increased secretion of IL-2 by non-Treg CD4^+^ T cells [88]. Our study showed increased FoxP3 expression and higher CD4^+^/CD8^+^ ratios in CD27^+^ tumor infiltrating lymphocytes surrounding CD70^+^ tumor cells in NSCLC patients. As such, CD70^+^ NSCLC cells could exhibit immune suppression through binding of CD27^+^ Tregs in the TME [9]. In addition to increased numbers, CD70-CD27 interaction also mediated enhanced survival of Tregs of CLL patients due to a decrease of pro-apoptotic Noxa and increase in anti-apoptotic Bcl-2, which resulted in a decreased sensitivity to drug-induced apoptosis [107]. In addition, the study of Yang et al. [108] reported an effect of CD70-CD27 on intratumoral Tregs. The authors found that expression of FoxP3 on intratumoral Tregs was (partially) induced by CD70^+^ lymphoma B cells and that these cells reduced the proliferation of infiltrating CD8^+^ T cells, revealing a role for CD70 expressing malignant cells in the development of intratumoral Tregs and immune suppression [108].

A recent study demonstrated that CD27 expression on human Tregs was closely correlated with suppression of CD4^+^ and CD8^+^ T cell proliferation, although the underlying mechanisms are still unclear [37]. Even though CD27 signaling has been shown to increase cell survival of both effector T cells and Tregs, their contribution has been shown to depend on the context and the predominance of each of these two cell types [109]. While in a pro-inflammatory environment, such as secondary lymph nodes, survival of effector T cells was enhanced upon CD27 signaling, increased survival of intratumoral Tregs was observed upon CD27 signaling in the context of a chronically inflamed and well-established tumor [109].

The CD70-CD27 interaction may also favor immune escape through the depletion of NK cells. The study of De Colvenaer et al. [110] used a CD70 transgenic mouse model in which all B cells constitutively express CD70 in order to study continuous triggering of CD27 on NK cells. Here, the authors showed that CD27 stimulation by CD70 resulted in reduced CD27 expression on NK cells as well as depletion of predominantly mature NK cells in vivo, which was partially due to increased apoptosis [110].

Another mechanism by which CD70 expressing tumor cells can exert immune suppression is by regulating immunosuppressive myeloid cells, such as macrophages. A recent study reported an association between CD70 expression and infiltration of CD163^+^ (a marker of M2 macrophages) tumor-associated macrophages (TAMs) in glioblastoma, suggesting a (in)direct role for CD70 in recruitment and/or activation of tumor promoting TAMs [84, 95, 111]. Here, they also showed that the presence of CD70 on tumor cells and CD163 on TAMs correlated with poor prognosis for glioblastoma patients [95].

Even when expression of CD70 is limited on tumor cells, it can find its way to sustain a tumor enhancing environment by hijacking other important players within the TME that contribute to the proliferative and invasive behavior of cancer, such as the cancer-associated fibroblasts (CAFs). These CAFs, present within the tumor margins and/or tumor mass, exist of a heterogeneous population that forms one of the most dominant components in the TME. In particular cancer types, such as colorectal carcinoma and pancreatic cell carcinoma, these CAFs contribute to oncogenesis and therapy resistance [112, 113]. We recently discovered a subset of CD70 expressing CAFs in specimens of colorectal carcinoma patients [114]. These CD70^high^ CAFs were shown to mediate immune suppression in vitro through increased numbers of CD4^+^ FoxP3^+^ CD25^+^ Tregs and IL-2 levels as opposed to the CD70^low^ CAF population, likely mitigated through CD27. In addition, while colorectal carcinoma cells alone showed no migratory ability, the co-culture with CD70^high^ CAFs exhibited a strong increase in migration. In line with the former, the study of Inoue et al. [115] observed an inferior prognosis in colorectal carcinoma patients with CD70^high^ CAFs [115]. The presence of CD70^+^ CAFs was also described in head and neck squamous cell carcinoma and since a role of CD70 on CAFs is still a very recent finding, it is very likely that other tumor types will follow [116].

**Fig. 3:**
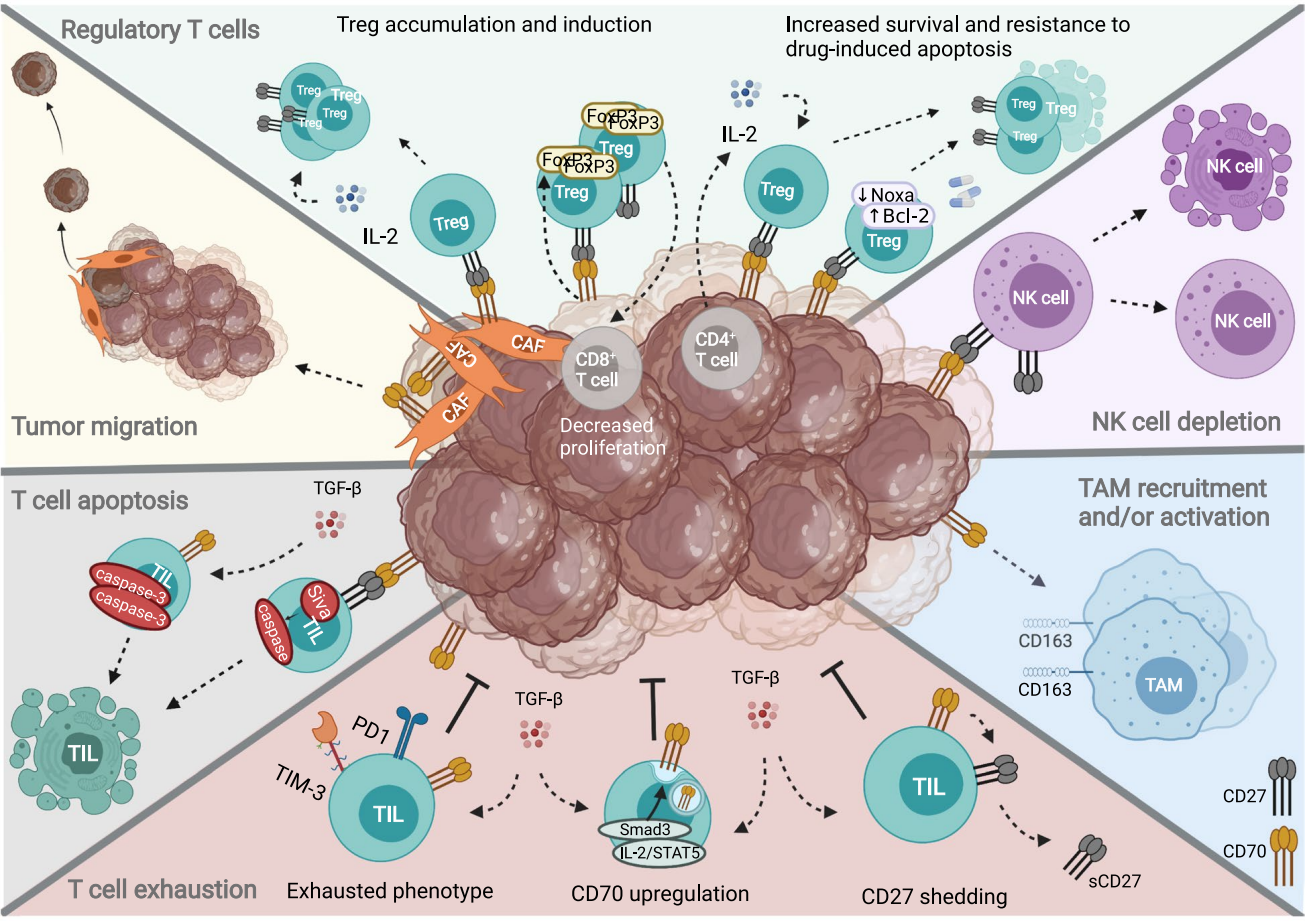
The CD70-CD27 axis and the tumor microenvironement. The CD70-CD27 axis facilitates immune evasion and suppression through different cell populations in the TME. The CD70-CD27 axis is reported to increase Treg survival and proliferation and T cell exhaustion and apoptosis. In addition, the axis has been linked to depletion of NK cells and migration/activation of TAMs. Moreover, expression of CD70 on CAFs can promote immune evasion through Treg accumulation and tumor migration and invasion. Figure created with BioRender.com. Abbreviations; Bcl2, B cell lymphoma 2; CAF, cancer-associated fibroblast; FoxP3, forkhead box P3; IL-2, interleukin 2; NK cell, natural killer cell; PD1, programmed cell death protein 1; sCD27, soluble CD27; Smad3, suppressor of mothers against decapentaplegic homolog 3; STAT5, signal transducer and activator of transcription 5; TAM, tumor-associated macrophage; TGF-β, transforming growth factor beta; TIL, tumor infiltrating lymphocyte; TIM-3, T cell immunoglobulin mucin-3; TME, tumor microenvironment; Treg, regulatory T cell

These findings underline the role of the CD70-CD27 axis in facilitating immune evasion through different cells of the TME. An overview of the CD70-CD27 axis and the TME in solid cancers is depicted in Fig. 3.

### Regulation of CD70 expression

So far, the exact underlying mechanisms of CD70^+^ tumor cells remains largely unknown, although several studies suggest that epigenetic alterations regulate CD70 expression. DNA demethylation of the *Tnfsf7* promotor gene has previously been suggested to upregulate CD70 on T cells in the context of autoimmune diseases [117]. Along these lines, in the context of cancer, hypermethylation of the promotor region downregulated *Tnfsf7* gene expression in an in vitro breast cancer MCF10 model [118]. The promotor site of the CD70 gene contains binding sites for transcription factors, such as AP-1, Sp1, NF-κB and AP-2. While Sp1 has been linked to CD70 upregulation, the other transcription factors might be implicated as well [45]. Apart from epigenetics, other factors have been reported to drive CD70 expression in response to changes in the TME, such as production of TGF-β and proinflammatory cytokines TNF-α and IFN-γ [49], stimulation of the CD40L-CD40 axis [47, 119, 120] and higher expression of hypoxia-related transcription factors HIF-α/β under hypoxia [90, 99, 100, 121]. Induction of CD70 expression has also been linked to cancer types caused by viral infections, such as Epstein-Barr virus and Human T-lymphotropic virus type 1 [73, 74, 78, 122, 123]. Finally, neutralization of endogenous IL-18 was reported to upregulate CD70 in stomach cancer [124].

### Targeting CD70

New insights into the tumor progressive role of CD70 further strengthen the rationale of exploiting CD70 in cancer patients, having the potential to (1) specifically eliminate the CD70 expressing cancer cell populations and (2) abrogate the tumor promoting mechanisms by the CD70-CD27 signaling axis, both in early stage and advanced disease. Since CD70 expression is absent during homeostasis, as opposed to CD27, it has great potential to exploit as a cancer-specific target. It should be noted that activated T/B cells can transiently express CD70 and thus, caution should be taken when evaluating anti-CD70 approaches given that off-target effects remain a challenge.

**Fig. 4:**
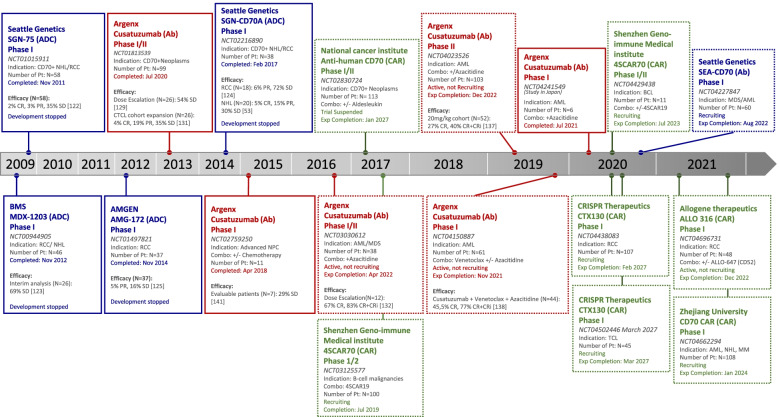
Overview of ongoing and completed clinical trials with CD70-targeting agents. Completed trials (solid line) and ongoing trials (dashed line) were sorted based on study start date. Antibody-drug conjugates (ADC, blue); Antibody (Ab, Red); Chimeric antigen receptor (CAR, Green). Efficacy data are depicted when available. Abbreviations: ADC, antibody drug conjugate: Ab, antibody; CAR, chimeric antigen receptor; CR, complete remission; PR, partial remission; SD, stable disease; CRi, CR with incomplete hematological recovery; NHL, non-Hodgkin lymphoma; CTCL, cutaneous T cell lymphoma; RCC, renal cell carcinoma; NPC, nasopharynx carcinoma; AML, acute myeloid leukemia; MDS, myelodysplastic syndrome; BCL, B cell lymphoma; MM, multiple myeloma; TCL, T cell lymphoma; Pt, patients; Exp, expected

As such, numerous promising therapeutic approaches targeting CD70 have been under investigation, both in preclinical and clinical settings with the aim of improving treatment outcomes of cancer patients. An overview of all strategies completed or currently undergoing clinical evaluation is shown in Fig. 4 (extensively reviewed in [125]).

#### Antibodies

Antibody-drug conjugates (ADCs) are monoclonal antibodies (mAbs) coupled to a cytotoxic agent, which enable specific binding of the target protein and internalization of the cytotoxic agent resulting in tumor cell killing. Several ADC compounds targeting CD70 have been developed and are undergoing clinical evaluation in hematological and solid cancers [56, 126–129]. Four different ADCs entered the clinic and all have completed phase I, of which development of two compounds, AMG 172 (Amgen) and MDX-1203 (Bristol-Myers Squibb), has stopped [127]. While both ADC compounds of Seattle Genetics (SGN-75 and SGN-CD70A) discontinued clinical testing due to toxicity reasons, the company’s non-fucosylated monoclonal antibody SEA-CD70 is currently undergoing clinical evaluation in a phase I study in patients with myeloid malignancies (NCT04227847).

Although ADCs targeting CD70 are a promising approach to mediate selective killing of the tumor cells, these agents rely on the extent of internalization, which can differ greatly among tumor types [130, 131].

Another form of antibody-mediated therapy is based on antibody-dependent cellular cytotoxicity (ADCC) relying on activating effector cells that carry the Fc receptor CD16 (FcγRIII) combined with blocking of the target protein. The most typical Fc-bearing receptor effector cells that are activated upon binding are the macrophages, although other effector cells, such as NK cells, γδ T cells and dendritic cells also mediate ADCC [132]. Cusatuzumab (ARGX-110) has next to complement-dependent cytotoxicity, enhanced ADCC properties due to increased binding to FcγRIIIa (as a consequence of afucosylation) [72, 133, 134]. Its safety profile has demonstrated to be favorable with no dose limiting toxicities in patients with advanced solid and hematological malignancies [133]. In AML, cusatuzumab efficiently eliminated LSCs in patients treated with hypomethylating agents whereas blocking the CD70-CD27 pathway induced differentiation in AML blasts and stem/progenitor cells [135]. One of the challenges that antibodies relying on ADCC are facing, is the inadequate activation and function of the immune effector cells, such as NK cells, since a great number of cancer patients have an impaired immune system [136].

#### CAR-therapy

Chimeric Antigen Receptor (CAR)-T cell therapy harnesses the patient’s own T cells to better recognize and eliminate the patient’s cancer cells by modifying these T cells to recognize tumor antigens in an HLA independent manner. CAR-T cell therapy has already achieved tremendous success in the treatment of hematological malignancies resulting in FDA/EMA approvals for CD19-targeting CAR-T cell compounds: Kymriah (DLBCL, ALL), Yescarta (DLBCL, follicular lymphoma), Breyanzi (DLBCL, follicular lymphoma), Tecartus (CML) and Abecma (MM) [137]. In addition to different CAR-therapies in preclinical development, currently, five anti-CD70 CAR-T based approaches, i.e., anti-hCD70 CAR (NCT02830724), 4SCAR70 (NCT03125577), CAR CD70 (NCT04662294), CTX130 (NCT04438083) and ALLO316 (NCT04696731), are being evaluated in phase I/II clinical trials in patients with hematological and solid malignancies, awaiting results regarding the safety profile and efficacy in these different cancer types. Despite its success in hematological malignancies, it remains challenging to treat solid tumors with this treatment modality due to multiple obstacles, such as trafficking of the transferred cells to the tumor site, penetration into the tumor and maintaining viability [138].

#### Combination regimens evaluated in clinical setting

Combination regimens with CD70 targeting agents have already been explored in clinical setting for certain cancer types. Preclinical research showed that azacitidine, a hypomethylating agent (HMA), upregulated CD70 on AML LSCs, making these malignant cells more susceptible to CD70 targeting [135]. Based on these preclinical findings, a phase I/II study was initiated, combining cusatuzumab with azacitidine, in previously untreated older patients with AML or high risk myelodysplastic (NCT03030612). Initial results from the phase I/II dose escalation demonstrate hematological responses in all patients with a complete remission in 67% (8/12) and complete remission with incomplete blood count recovery in 17% (2/12) of patients [139]. Additionally, no dose-limiting toxicities were reported and the maximum tolerated dose of cusatuzumab was not reached. These promising results have led to a randomized phase II trial in newly diagnosed patients with AML, unfit for intensive chemotherapy, combining azacitidine with 10 mg/kg or 20 mg/kg of cusatuzumab (NCT04023526) [140].

Another promising regimen that recently entered the clinic is a combination of cusatuzumab and venetoclax (+/− azacitidine) (NCT04150887) [141]. BCL-2 plays an important role in the survival and persistence of AML blasts by sequestering pro-apoptotic BAX. Venetoclax is a selective inhibitor of BCL-2 which results in the release of BAX leading to mitochondrial outer membrane permeabilization and thus apoptosis of LSCs. Although venetoclax demonstrates promising activity in elderly or chemotherapy-ineligible AML patients, successfully eliminating all LSCs remains a major challenge in the effective treatment of AML [142]. Similar to venetoclax, anti-CD70 therapy targets AML LSCs, though by a different mechanism i.e., inhibition of LSC proliferation, stimulation of their differentiation into myeloid cells and effector function-mediated cell killing [135]. Hence, combining two complementary agents that both target AML LSCs could create additive/synergistic anti-tumor effects and thus minimize drug resistance. Preclinical data could already demonstrate such synergism between cusatuzumab and venetoclax (+/−HMA) on LSCs [143]. Finally, a combination of cusatuzumab with radiotherapy and/or chemotherapy was evaluated in a phase I study in patients with nasopharyngeal carcinoma (NCT02759250). Patients that received prior radiotherapy and/or chemotherapy had longer progression-free survival compared to the cohort of patients that received monotherapy, although it should be noted that the size was too limited for any conclusive statements [144]. Currently there are no new clinical trials evaluating combinatorial approaches of anti-CD70 therapy with either chemotherapy or radiotherapy in solid cancers.

The combination therapy of CAR targeting CD19 and CD70 has already entered the clinic in patients who have relapsed/refractory B cell malignancies after chemotherapy (NCT03125577, NCT04429438). While the clinical study is currently still ongoing, preliminary results from a patient with refractory and relapsed primary central nervous system lymphoma demonstrated long-term disease free survival without inducing severe cytokine release syndrome and CART cell-related encephalopathy syndrome [145].

#### Combination regimens evaluated in pre-clinical setting

Next to the combinatorial regimens with CD70-targeting therapy that have entered the clinic, other innovative regimens are being explored in a preclinical setting. One of these studies explored the combination of anti-CD70 therapy and a tyrosine kinase inhibitor directed against BCR-ABL1 in CML. Here, BCR-ABL1 kinase is constitutively active in the majority of CML patients and tyrosine kinase inhibitors, targeting the oncogenic product BCR-ABL, are an effective treatment strategy in CML patients [146]. However, similar to AML, disease-initiating LSCs form a major challenge in the treatment of CML due to persistence of drug-resistant LSCs. The tyrosine kinase inhibitor, imatinib, was found to mediate gene expression changes of SP1 (upregulation) and DNMT1 (downregulation) resulting in demethylation of the CD70 promotor in CML cells and thus upregulated CD70 expression and compensatory Wnt signaling in CML cells [45]. In this study, the authors showed that dual targeting of BCR-ABL1 and CD70, synergistically eradicated CML LSCs due to more effective prevention of the Wnt pathway activation and rendering CML LSCs more susceptible to cell killing with anti-CD70 immunotherapy [45]. Thus, dual targeting of BCR-ABL1 and CD70 eliminates LSCs and may overcome therapy resistance.

Different studies report an interesting regimen of CD70-targeting therapy combined with chemotherapy. In NSCLC, it was shown that treatment with cisplatin increased CD70 expression, both on mRNA and protein level. Interestingly, the combination of cisplatin and the anti-CD70 agent cusatuzumab led to synergistic killing of NSCLC cell lines in vitro [101]. In ovarian carcinoma, CD70 expression on tumor cells was associated with clinical resistance to cisplatin [85]. Here, authors showed that CD70 targeting antibodies could inhibit proliferation of chemo-resistant tumor cells. As such, combining chemo and treatment with anti-CD70 antibodies could be used to overcome resistance to chemotherapy. Finally, it was demonstrated that radiotherapy, similar to what was seen with chemotherapy, could increase membrane CD70 expression in glioma, leukemia and lymphoma [35, 147, 148]. As such, combination strategies of anti-CD70 antibodies and chemotherapy/radiotherapy could render malignant cells more susceptible to CD70 targeted therapy.

#### The different side of the coin

In this review on the CD70-CD27 axis, we focus on immunotherapeutic strategies aiming at eradicating the tumor cells by specifically targeting tumor-associated CD70 overexpression, thereby abrogating the immune suppressive effects driven by chronic stimulation of the CD70-CD27 axis. However, it is noteworthy to mention that other therapeutic strategies are under investigation with the focus on stimulating the CD27 signaling pathway to enhance CTL responses, including the agonistic IgG1 anti-CD27 antibody, CDX-1127/varlilumab and TriMix, a monocyte-derived DC vaccine is activated through electroporation with mRNA encoding CD40L, CD70 and active TLR4 [149, 150]. The identification of biomarkers and stratification concepts are important to select the appropriate therapeutic strategy of either inhibiting CD70 and/or CD27 activity, based on the underlying dominant functions, that ensures the best fit for the patient. Thus, targeting strategies of both sides of the CD70-CD27 axis may be exploited depending on the cancer type and its TME.

## Conclusions

Combinatorial approaches with anti-CD70 targeting therapies have proven their potential in both preclinical and clinical settings. Until now, mono- and combinatorial therapies have mainly been explored in AML, yet other tumor types might benefit from this approach as well. Research in the field has led to more insights into the underlying molecular mechanisms of the tumor promoting and immune evasive role of the CD70-CD27 axis in oncology. Therefore, in addition to existing approaches focused on targeting CD70, strategies that inhibit the signaling pathways involved in the CD70-CD27 axis might become promising novel therapeutic alternatives in the future. Further preclinical research and clinical evaluation of CD70 targeting strategies will provide new insights into the mechanisms and effects of CD70 and might pave the way towards novel treatment options in the field of oncology.

## Data Availability

Not applicable.

## Abbreviations

FDA: Food and Drug Administration
EMA: European Medicines Agency
CAR: Chimeric antigen-receptor
TNF: Tumor necrosis factor
sCD27: Soluble CD27
TME: Tumor microenvironment
HSCs: Hematopoietic stem cells
CD27^+^: CD27 positive
CD70^+^: CD70 positive
Tregs: Regulatory T cells
NK: Natural killer
NF- κB: Nuclear factor kappa B
TRAF2/5: TNF receptor-associated factors 2 and 5
PI3K: phosphoinositide 3-kinase
MEK: Mitogen-activated protein kinase kinase
LSCs: Leukemic stem cells
AML: Acute myeloid leukemia
CML: Chronic myeloid leukemia
B-ALL: B cell acute lymphoblastic leukemia
B-CLL: B cell chronic lymphocytic leukemia
NHL: Non-Hodgkin lymphoma
AP-1: Activator protein 1
ß-cat: ß-catenin
JAK/STAT: Janus kinase-signal transducer and activator of transcription
MAPK: Mitogen-activated protein kinase
PKC: Protein kinase C
RhoE: Ras homologous E
SOX2: Sex determining region Y-box 2
Vim: Vimentin
CSCs: Cancer stem cells
NSCLC: Non-small cell lung cancer
EMT: Epithelial to mesenchymal transition
TGF-β: Transforming growth factor beta
TAMs: Tumor-associated macrophages
CAFs: Cancer-associated fibroblasts
Bcl-2: B cell lymphoma 2
FoxP3: Forkhead box P3
PD1: Programmed cell death protein 1
Smad3: Suppressor of mothers against decapentaplegic homolog 3
STAT5: Signal transducer and activator of transcription 5
TIL: Tumor infiltrating lymphocyte
TIM-3: T cell immunoglobulin mucin-3
CR: Complete remission
CRi: Complete remission with incomplete hematological recovery
PR: Partial remission
SD: Stable disease
CTCL: Cutaneous T cell lymphoma
RCC: Renal cell carcinoma
BCL: B cell lymphoma
ADCs: Antibody drug conjugates
ADCC: Antibody-dependent cell-mediated cytotoxicity
ADCP: Antibody-dependent cellular phagocytosis
Ab: Antibody
mAbs: Monoclonal antibodies
NPC: Nasopharyngeal carcinoma
MDS: Myelodysplastic syndrome
DLBCL: Diffuse large B cell lymphoma
HMA: Hypomethylating agent

## References

[1] Chen DS, Mellman I (2013). Oncology meets immunology: the cancer-immunity cycle. Immunity..

[2] Dammeijer F, Lau SP, van Eijck CHJ, van der Burg SH, Aerts JGJV (2017). Rationally combining immunotherapies to improve efficacy of immune checkpoint blockade in solid tumors. Cytokine Growth Factor Rev.

[3] Carrega P, Loiacono F, Di Carlo E, Scaramuccia A, Mora M, Conte R (2015). NCR+ILC3 concentrate in human lung cancer and associate with intratumoral lymphoid structures. Nat Commun.

[4] Sterner RC, Sterner RM (2021). CAR-T cell therapy: current limitations and potential strategies. Blood Cancer J.

[5] Tang T, Huang X, Zhang G, Hong Z, Bai X, Liang T (2021). Advantages of targeting the tumor immune microenvironment over blocking immune checkpoint in cancer immunotherapy. Signal Transduct Target Ther.

[6] Qu J, Jiang M, Wang L, Zhao D, Qin K, Wang Y (2020). Mechanism and potential predictive biomarkers of immune checkpoint inhibitors in NSCLC. Biomed Pharmacother.

[7] Flieswasser T, Camara-Clayette V, Danu A, Bosq J, Ribrag V, Zabrocki P (2019). Screening a broad range of solid and haematological tumour types for CD70 expression using a uniform IHC methodology as potential patient stratification method. Cancers (Basel).

[8] Jacobs J, Deschoolmeester V, Zwaenepoel K, Rolfo C, Silence K, Rottey S (2015). CD70: An emerging target in cancer immunotherapy. Pharmacol Ther.

[9] Jacobs J, Zwaenepoel K, Rolfo C, Van den Bossche J, Deben C, Silence K (2015). Unlocking the potential of CD70 as a novel immunotherapeutic target for non-small cell lung cancer. Oncotarget..

[10] van Oers MH, Pals ST, Evers LM, van der Schoot CE, Koopman G, Bonfrer JM (1993). Expression and release of CD27 in human B-cell malignancies. Blood..

[11] Kok M, Bonfrer JM, Korse CM, de Jong D, Kersten MJ (2003). Serum soluble CD27, but not thymidine kinase, is an independent prognostic factor for outcome in indolent non-Hodgkin’s lymphoma. Tumor Biol.

[12] Riether C, Schurch CM, Buhrer ED, Hinterbrandner M, Huguenin A-L, Hoepner S (2017). CD70/CD27 signaling promotes blast stemness and is a viable therapeutic target in acute myeloid leukemia. J Exp Med.

[13] Kashima J, Okuma Y, Hosomi Y, Hishima T (2019). High serum soluble CD27 level correlates with poor performance status and reduced survival in patients with advanced lung Cancer. Oncology..

[14] Goto N, Tsurumi H, Takemura M, Kanemura N, Kasahara S, Hara T (2012). Serum soluble CD27 level is associated with outcome in patients with diffuse large B-cell lymphoma treated with rituximab, cyclophosphamide, doxorubicin, vincristine and prednisolone. Leuk Lymphoma.

[15] Riether C, Schürch CM, Ochsenbein AF (2015). Regulation of hematopoietic and leukemic stem cells by the immune system. Cell Death Differ.

[16] Wiesmann A, Phillips RL, Mojica M, Pierce LJ, Searles AE, Spangrude GJ (2000). Expression of CD27 on murine hematopoietic stem and progenitor cells. Immunity..

[17] Nowlan B, Williams ED, Doran MR, Levesque J-P (2020). CD27, CD201, FLT3, CD48, and CD150 cell surface staining identifies long-term mouse hematopoietic stem cells in immunodeficient non-obese diabetic severe combined immune deficient-derived strains. Haematologica..

[18] Vazquez SE, Inlay MA, Serwold T (2015). CD201 and CD27 identify hematopoietic stem and progenitor cells across multiple murine strains independently of kit and Sca-1. Exp Hematol.

[19] Serwold T, Richie Ehrlich LI, Weissman IL (2009). Reductive isolation from bone marrow and blood implicates common lymphoid progenitors as the major source of thymopoiesis. Blood..

[20] Nolte MA, Arens R, van Os R, van Oosterwijk M, Hooibrink B, van Lier RAW (2005). Immune activation modulates hematopoiesis through interactions between CD27 and CD70. Nat Immunol.

[21] Schürch C, Riether C, Matter MS, Tzankov A, Ochsenbein AF (2012). CD27 signaling on chronic myelogenous leukemia stem cells activates Wnt target genes and promotes disease progression. J Clin Invest.

[22] Nilsson A, de Milito A, Mowafi F, Winberg G, Bjork O, Wolpert EZ (2005). Expression of CD27-CD70 on early B cell progenitors in the bone marrow: implication for diagnosis and therapy of childhood ALL. Exp Hematol.

[23] Vaskova M, Fronkova E, Starkova J, Kalina T, Mejstrikova E, Hrusak O (2008). CD44 and CD27 delineate B-precursor stages with different recombination status and with an uneven distribution in nonmalignant and malignant hematopoiesis. Tissue Antigens.

[24] McWilliams L, Su K-Y, Liang X, Liao D, Floyd S, Amos J (2013). The human fetal lymphocyte lineage: identification by CD27 and LIN28B expression in B cell progenitors. J Leukoc Biol.

[25] Jung J, Choe J, Li L, Choi YS (2000). Regulation of CD27 expression in the course of germinal center B cell differentiation: the pivotal role of IL-10. Eur J Immunol.

[26] Wu Y-CB, Kipling D, Dunn-Walters DK (2011). The relationship between CD27 negative and positive B cell populations in human peripheral blood. Front Immunol.

[27] Lens SMA, De JR, Hintzen RQ, Koopman G, Van Lier RAW, Van Oers RHJ (1995). CD27–CD70 interaction: Unravelling its implication in Normal and neoplastic B-cell growth. Leuk Lymphoma.

[28] Lens SMA, De Jong R, Hooibrink B, Koopman G, Pals ST, van Oers MHJ (1996). Phenotype and function of human B cells expressing CD70 (CD27 ligand). Eur J Immunol.

[29] Hintzen RQ, Lens SMA, Koopman G, Pals ST, Spits H, van Lier RAW (1994). CD70 represents the human ligand for CD27. Int Immunol.

[30] Martorell J, Rojo I, Vilella R, Martinez-Caceres E, Vives J (1990). CD27 induction on thymocytes. J Immunol.

[31] van Lier RA, Borst J, Vroom TM, Klein H, Van Mourik P, Zeijlemaker WP (1987). Tissue distribution and biochemical and functional properties of Tp55 (CD27), a novel T cell differentiation antigen. J Immunol.

[32] Bautista JL, Cramer NT, Miller CN, Chavez J, Berrios DI, Byrnes LE (2021). Single-cell transcriptional profiling of human thymic stroma uncovers novel cellular heterogeneity in the thymic medulla. Nat Commun.

[33] Hendriks J, Gravestein LA, Tesselaar K, van Lier RAW, Schumacher TNM, Borst J (2000). CD27 is required for generation and long-term maintenance of T cell immunity. Nat Immunol.

[34] Ribot JC, DeBarros A, Pang DJ, Neves JF, Peperzak V, Roberts SJ (2009). CD27 is a thymic determinant of the balance between interferon-γ- and interleukin 17–producing γδ T cell subsets. Nat Immunol.

[35] Hintzen RQ, Lens SM, Beckmann MP, Goodwin RG, Lynch D, van Lier RA (1994). Characterization of the human CD27 ligand, a novel member of the TNF gene family. J Immunol.

[36] Nolte MA, van Olffen RW, van Gisbergen KPJM, van Lier RAW (2009). Timing and tuning of CD27-CD70 interactions: the impact of signal strength in setting the balance between adaptive responses and immunopathology. Immunol Rev.

[37] Arroyo Hornero R, Georgiadis C, Hua P, Trzupek D, He L-Z, Qasim W (2020). CD70 expression determines the therapeutic efficacy of expanded human regulatory T cells. Commun Biol.

[38] Schiött A, Lindstedt M, Johansson-Lindbom B, Roggen E, Borrebaeck CAK (2004). CD27- CD4+ memory T cells define a differentiated memory population at both the functional and transcriptional levels. Immunology..

[39] Vossen MTM, Matmati M, Hertoghs KML, Baars PA, Gent M-R, Leclercq G (2008). CD27 defines phenotypically and functionally different human NK cell subsets. J Immunol.

[40] Yang FC, Agematsu K, Nakazawa T, Mori T, Ito S, Kobata T, Morimoto C, Komiyama A (1996). CD27/CD70 interaction directly induces natural killer cell killing activity. Immunology..

[41] Arens R, Nolte MA, Tesselaar K, Heemskerk B, Reedquist KA, van Lier RAW (2004). Signaling through CD70 regulates B cell activation and IgG production. J Immunol.

[42] Al Sayed MF, Ruckstuhl CA, Hilmenyuk T, Claus C, Bourquin J-P, Bornhauser BC (2017). CD70 reverse signaling enhances NK cell function and immunosurveillance in CD27-expressing B-cell malignancies. Blood..

[43] Shinozaki K, Yasui K, Agematsu K (2001). Direct B/B-cell interactions in immunoglobulin synthesis. Clin Exp Immunol.

[44] García P, de Heredia AB, Bellón T, Carpio E, Llano M, Caparrós E (2004). Signalling via CD70, a member of the TNF family, regulates T cell functions. J Leukoc Biol.

[45] Riether C, Schurch CM, Flury C, Hinterbrandner M, Druck L, Huguenin A-L (2015). Tyrosine kinase inhibitor-induced CD70 expression mediates drug resistance in leukemia stem cells by activating Wnt signaling. Sci Transl Med.

[46] Troeger A, Glouchkova L, Escherich G, Siepermann M, Hanenberg H, Janka-Schaub G (2008). Reduced expression and defective modulation of TNF receptor/ligand family molecules on proB-ALL blasts. Klin Pädiatrie.

[47] Glouchkova L, Ackermann B, Zibert A, Meisel R, Siepermann M, Janka-Schaub GE (2009). The CD70/CD27 pathway is critical for stimulation of an effective cytotoxic T cell response against B cell precursor acute lymphoblastic leukemia. J Immunol.

[48] Pálóczi K, Pócsik É, Mihalik R, Benczur M, Demeter J, Solti V (1990). Detection of activation antigens on chronic lymphocytic Leukaemia cells. Leuk Lymphoma.

[49] Ranheim EA, Cantwell MJ, Kipps TJ (1995). Expression of CD27 and its ligand, CD70, on chronic lymphocytic leukemia B cells. Blood..

[50] Trentin L, Zambello R, Sancetta R, Facco M, Cerutti A, Perin A (1997). B lymphocytes from patients with chronic lymphoproliferative disorders are equipped with different costimulatory molecules. Cancer Res.

[51] Lens SM, Drillenburg P, den Drijver BF, van Schijndel G, Pals ST, van Lier RA (1999). Aberrant expression and reverse signalling of CD70 on malignant B cells. Br J Haematol.

[52] Vetrie D, Helgason GV, Copland M (2020). The leukaemia stem cell: similarities, differences and clinical prospects in CML and AML. Nat Rev Cancer.

[53] Martinez-Climent JA, Fontan L, Gascoyne RD, Siebert R, Prosper F (2010). Lymphoma stem cells: enough evidence to support their existence?. Haematologica..

[54] Jones RJ, Gocke CD, Kasamon YL, Miller CB, Perkins B, Barber JP (2009). Circulating clonotypic B cells in classic Hodgkin lymphoma. Blood..

[55] Matsui W, Wang Q, Barber JP, Brennan S, Smith BD, Borrello I (2008). Clonogenic multiple myeloma progenitors, stem cell properties, and drug resistance. Cancer Res.

[56] Phillips T, Barr PM, Park SI, Kolibaba K, Caimi PF, Chhabra S (2019). A phase 1 trial of SGN-CD70A in patients with CD70-positive diffuse large B cell lymphoma and mantle cell lymphoma. Investig New Drugs.

[57] Dong HY, Shahsafaei A, Dorfman DM (2002). CD148 and CD27 are expressed in B cell lymphomas derived from both memory and Naïve B cells. Leuk Lymphoma.

[58] McEarchern JA, Smith LM, McDonagh CF, Klussman K, Gordon KA, Morris-Tilden CA (2008). Preclinical characterization of SGN-70, a humanized antibody directed against CD70. Clin Cancer Res.

[59] Zhu Y, Hollmén J, Räty R, Aalto Y, Nagy B, Elonen E (2002). Investigatory and analytical approaches to differential gene expression profiling in mantle cell lymphoma. Br J Haematol.

[60] Ho AW, Hatjiharissi E, Ciccarelli BT, Branagan AR, Hunter ZR, Leleu X (2008). CD27-CD70 interactions in the pathogenesis of Waldenström macroglobulinemia. Blood..

[61] Bertrand P, Maingonnat C, Penther D, Guney S, Ruminy P, Picquenot JM (2013). The costimulatory molecule CD70 is regulated by distinct molecular mechanisms and is associated with overall survival in diffuse large B-cell lymphoma. Genes Chromosom Cancer.

[62] McEarchern JA, Oflazoglu E, Francisco L, McDonagh CF, Gordon KA, Stone I (2007). Engineered anti-CD70 antibody with multiple effector functions exhibits in vitro and in vivo antitumor activities. Blood..

[63] Stein H, Herbst H, Anagnostopoulos I, Niedobitek G, Dallenbach F, Kratzsch H-C (1991). The nature of Hodgkin and reed-Sternberg cells, their association with EBV, and their relationship to anaplastic large-cell lymphoma. Ann Oncol.

[64] Guikema JEJ, Hovenga S, Vellenga E, Conradie JJ, Abdulahad WH, Bekkema R (2003). CD27 is heterogeneously expressed in multiple myeloma: low CD27 expression in patients with high-risk disease. Br J Haematol.

[65] Katayama Y, Sakai A, Oue N, Asaoku H, Otsuki T, Shiomomura T (2003). A possible role for the loss of CD27-CD70 interaction in myelomagenesis. Br J Haematol.

[66] Guikema JEJ, Vellenga E, Abdulahad WH, Hovenga S, Bos NA (2004). CD27-triggering on primary plasma cell leukaemia cells has anti-apoptotic effects involving mitogen activated protein kinases. Br J Haematol.

[67] Pileri S, Falini B, Delsol G, Stein H, Baglioni P, Poggi S (1990). Lymphohistiocytic T-cell lymphoma (anaplastic large cell lymphoma CD30+/Ki-1 + with a high content of reactive histiocytes). Histopathology..

[68] Hultberg A, Gandini D, Bagot M, Maerevoet M, Zwanenpoel K, De Winne K (2017). CD70 expression in cutaneous T cell lumphoma (CTCL) patients and mechanisms of action of ARGX-110 in skin: histopathological and clinical data. Hematol Oncol.

[69] Berti E, Cerri A, Cavicchini S, Delia D, Soligo D, Alessi E (1991). Primary cutaneous γ/δ T-cell lymphoma presenting as disseminated Pagetoid Reticulosis. J Invest Dermatol.

[70] Campbell JJ, Clark RA, Watanabe R, Kupper TS (2010). Sézary syndrome and mycosis fungoides arise from distinct T-cell subsets: a biologic rationale for their distinct clinical behaviors. Blood..

[71] Van DR, Dijkman R, Vermeer MH, Out-luiting JJ, Van Der R-h EMH, Willemze R (2004). Aberrant expression of the tyrosine kinase receptor EphA4 and the transcription factor twist in Sézary syndrome identified by gene expression analysis. Cancer Res.

[72] Leupin N, Zinzani PL, Morschhauser F, Dalle S, Maerevoet M, Michot J, et al. Cusatuzumab for treatment of CD70-positive relapsed or refractory cutaneous T-cell lymphoma. Cancer. 2021. 10.1002/cncr.34005. 10.1002/cncr.34005 34726773

[73] Baba M, Okamoto M, Hamasaki T, Horai S, Wang X, Ito Y (2008). Highly enhanced expression of CD70 on human T-Lymphotropic virus type 1-carrying T-cell lines and adult T-cell leukemia cells. J Virol.

[74] Masamoto I, Yoshimitsu M, Kuroki A, Horai S, Ezinne CC, Kozako T (2016). Clinical significance of CD70 expression on T cells in human T-lymphotropic virus type-1 carriers and adult T cell leukemia/ lymphoma patients. Leuk Lymphoma.

[75] Shao H, Yuan CM, Xi L, Raffeld M, Morris JC, Janik JE (2010). Minimal residual disease detection by flow Cytometry in adult T-cell leukemia/lymphoma. Am J Clin Pathol.

[76] Yoshino K, Kishibe K, Nagato T, Ueda S, Komabayashi Y, Takahara M (2013). Expression of CD70 in nasal natural killer/T cell lymphoma cell lines and patients; its role for cell proliferation through binding to soluble CD27. Br J Haematol.

[77] Zheng M, Yu L, Hu J, Zhang Z, Wang H, Lu D (2020). Efficacy of B7-H3-redirected BiTE and CAR-T immunotherapies against Extranodal nasal natural killer/T cell lymphoma. Transl Oncol.

[78] Shaffer DR, Sheehan AM, Yi Z, Rodgers CC, Bollard CM, Brenner MK (2012). Aggressive peripheral CD70-positive t-cell lymphoma associated with severe chronic active EBV infection. Pediatr Blood Cancer.

[79] Shaffer DR, Savoldo B, Yi Z, Chow KKH, Kakarla S, Spencer DM (2011). T cells redirected against CD70 for the immunotherapy of CD70-positive malignancies. Blood..

[80] Kaufmann Y, Amariglio N, Rosenthal E, Hirsch YJ, Many A, Rechavi G (2005). Proliferation response of leukemic cells to CD70 ligation oscillates with recurrent remission and relapse in a Low-grade lymphoma. J Immunol.

[81] Erlichman B, Zack HO (1999). CD27 signals through PKC in human B cell lymphomas. Cytokine..

[82] Petrau C, Cornic M, Bertrand P, Maingonnat C, Marchand V, Picquenot J-M (2014). CD70: a potential target in breast Cancer?. J Cancer.

[83] Jilaveanu LB, Sznol J, Aziz SA, Duchen D, Kluger HM, Camp RL (2012). CD70 expression patterns in renal cell carcinoma. Hum Pathol.

[84] Jin L, Ge H, Long Y, Yang C, Chang YE, Mu L (2018). CD70, a novel target of CAR T-cell therapy for gliomas. Neuro-Oncology.

[85] Liu N, Sheng X, Liu Y, Zhang X, Yu J (2013). Increased CD70 expression is associated with clinical resistance to cisplatin-based chemotherapy and poor survival in advanced ovarian carcinomas. Onco Targets Ther.

[86] Inaguma S, Lasota J, Czapiewski P, Langfort R, Rys J, Szpor J (2020). CD70 expression correlates with a worse prognosis in malignant pleural mesothelioma patients via immune evasion and enhanced invasiveness. J Pathol.

[87] Ryan MC, Kostner H, Gordon KA, Duniho S, Sutherland MK, Yu C (2010). Targeting pancreatic and ovarian carcinomas using the auristatin-based anti-CD70 antibody-drug conjugate SGN-75. Br J Cancer.

[88] Claus C, Riether C, Schurch C, Matter MS, Hilmenyuk T, Ochsenbein AF (2012). CD27 signaling increases the frequency of regulatory T cells and promotes tumor growth. Cancer Res.

[89] Diegmann J, Junker K, Loncarevic IF, Michel S, Schimmel B, von Eggeling F (2006). Immune escape for renal cell carcinoma: CD70 mediates apoptosis in lymphocytes. Neoplasia..

[90] Yang Z-Z, Grote DM, Xiu B, Ziesmer SC, Price-Troska TL, Hodge LS (2014). TGF-beta upregulates CD70 expression and induces exhaustion of effector memory T cells in B-cell non-Hodgkin’s lymphoma. Leukemia..

[91] Pich C, Sarrabayrouse G, Teiti I, Mariame B, Rochaix P, Lamant L (2016). Melanoma-expressed CD70 is involved in invasion and metastasis. Br J Cancer.

[92] Yang L, Shi P, Zhao G, Xu J, Peng W, Zhang J (2020). Targeting cancer stem cell pathways for cancer therapy. Signal Transduct Target Ther.

[93] Prager BC, Xie Q, Bao S, Rich JN (2019). Cancer stem cells: the architects of the tumor ecosystem. Cell Stem Cell.

[94] Liu L, Yin B, Yi Z, Liu X, Hu Z, Gao W (2018). Breast cancer stem cells characterized by CD70 expression preferentially metastasize to the lungs. Breast Cancer.

[95] Ge H, Mu L, Jin L, Yang C, Chang YE, Long Y (2017). Tumor associated CD70 expression is involved in promoting tumor migration and macrophage infiltration in GBM. Int J Cancer.

[96] Ortiz-Cuaran S, Swalduz A, Albaret MA, Menetrier-Caux C, Haddad V, Paré A (2016). CD70 immune checkpoint ligand is associated with the epithelial-to-mesenchymal transition in non-small cell lung cancer. Proceedings of the 107th Annual Meeting of the American Association for Cancer Research; 2016 Apr 16–20; New Orleans, LA, Philadelphia (PA). AACR Cancer Res.

[97] Nakamura K, Sho M, Akahori T, Nishiwada S, Kunishige T, Nakagawa K (2021). Clinical relevance of CD70 expression in resected pancreatic cancer: prognostic value and therapeutic potential. Pancreatology..

[98] Tse SW, Tan CF, Park JE, Gnanasekaran J, Gupta N, Low JK (2020). Microenvironmental hypoxia induces dynamic changes in lung Cancer synthesis and secretion of extracellular vesicles. Cancers (Basel).

[99] Kitajima S, Lee KL, Fujioka M, Sun W, You J, Chia GS (2018). Hypoxia-inducible factor-2 alpha up-regulates CD70 under hypoxia and enhances anchorage-independent growth and aggressiveness in cancer cells. Oncotarget..

[100] Ruf M, Mittmann C, Nowicka AM, Hartmann A, Hermanns T, Poyet C (2015). pVHL/HIF-regulated CD70 expression is associated with infiltration of CD27+ lymphocytes and increased serum levels of soluble CD27 in clear cell renal cell carcinoma. Clin Cancer Res.

[101] Jacobs J, Deschoolmeester V, Rolfo C, Zwaenepoel K, Van Den BJ, Deben C (2017). Preclinical data on the combination of cisplatin and anti-CD70 therapy in non-small cell lung cancer as an excellent match in the era of combination therapy. Oncotarget..

[102] Wischhusen J, Jung G, Radovanovic I, Beier C, Steinbach JP, Rimner A (2002). Identification of CD70-mediated apoptosis of immune effector cells as a novel immune escape pathway of human glioblastoma. Cancer Res.

[103] Aulwurm S, Wischhusen J, Friese M, Borst J, Weller M (2006). Immune stimulatory effects of CD70 override CD70-mediated immune cell apoptosis in rodent glioma models and confer long-lasting antiglioma immunity in vivo. Int J Cancer.

[104] Yoon Y, Ao Z, Cheng Y, Schlossman SF, Prasad KV (1999). Murine Siva-1 and Siva-2, alternate splice forms of the mouse Siva gene, both bind to CD27 but differentially transduce apoptosis. Oncogene..

[105] Prasad KV, Ao Z, Yoon Y, Wu MX, Rizk M, Jacquot S (1997). CD27, a member of the tumor necrosis factor receptor family, induces apoptosis and binds to Siva, a proapoptotic protein. Proc Natl Acad Sci U S A.

[106] Wang QJ, Hanada K, Robbins PF, Li YF, Yang JC (2012). Distinctive features of the differentiated phenotype and infiltration of tumor-reactive lymphocytes in clear cell renal cell carcinoma. Cancer Res.

[107] Jak M, Mous R, Remmerswaal EBM, Spijker R, Jaspers A, Yagüe A (2009). Enhanced formation and survival of CD4 + CD25 hi Foxp3 + T-cells in chronic lymphocytic leukemia. Leuk Lymphoma.

[108] Yang Z, Novak AJ, Ziesmer SC, Witzig TE, Ansell SM (2017). CD70+ non-Hodgkin lymphoma B cells induce Foxp3 expression and regulatory function in intratumoral CD4+CD25 T cells. Blood..

[109] Riether C, Schurch C, Ochsenbein AF (2012). Modulating CD27 signaling to treat cancer. Oncoimmunology..

[110] De Colvenaer V, Taveirne S, Hamann J, de Bruin AM, De Smedt M, Taghon T (2010). Continuous CD27 triggering in vivo strongly reduces NK cell numbers. Eur J Immunol.

[111] Hu JM, Liu K, Liu JH, Jiang XL, Wang XL, Chen YZ (2017). CD163 as a marker of M2 macrophage, contribute to predicte aggressiveness and prognosis of Kazakh esophageal squamous cell carcinoma. Oncotarget..

[112] Liu T, Han C, Wang S, Fang P, Ma Z, Xu L (2019). Cancer-associated fibroblasts: an emerging target of anti-cancer immunotherapy. J Hematol Oncol.

[113] Domen A, Quatannens D, Zanivan S, Deben C, Van Audenaerde J, Smits E (2021). Cancer-associated fibroblasts as a common orchestrator of therapy resistance in lung and pancreatic Cancer. Cancers (Basel).

[114] Jacobs J, Deschoolmeester V, Zwaenepoel K, Flieswasser T, Deben C, Van den Bossche J (2018). Unveiling a CD70-positive subset of cancer-associated fibroblasts marked by pro-migratory activity and thriving regulatory T cell accumulation. Oncoimmunology..

[115] Inoue S, Ito H, Tsunoda T, Murakami H, Ebi M, Ogasawara N (2019). CD70 expression in tumor-associated fibroblasts predicts worse survival in colorectal cancer patients. Virchows Arch.

[116] De Meulenaere A, Vermassen T, Aspeslagh S, Zwaenepoel K, Deron P, Duprez F (2016). CD70 expression and its correlation with Clinicopathological variables in squamous cell carcinoma of the head and neck. Pathobiology..

[117] Lu Q, Wu A, Richardson BC (2005). Demethylation of the same promoter sequence increases CD70 expression in lupus T cells and T cells treated with lupus-inducing drugs. J Immunol.

[118] Yu SE, Park SH, Jang YK (2010). Epigenetic silencing of TNFSF7 (CD70) by DNA methylation during progression to breast cancer. Mol Cell.

[119] Kato K, Cantwell MJ, Sharma S, Kipps TJ (1998). Gene transfer of CD40-ligand induces autologous immune recognition of chronic lymphocytic leukemia B cells. J Clin Invest.

[120] Troeger A, Glouchkova L, Ackermann B, Escherich G, Hanenberg H, Janka G (2014). Significantly increased CD70 up regulation on TEL-AML positive B cell precursor acute lymphoblastic leukemia cells following CD40 stimulation. Klin Padiatr.

[121] Holmquist-Mengelbier L, Fredlund E, Lofstedt T, Noguera R, Navarro S, Nilsson H (2006). Recruitment of HIF-1alpha and HIF-2alpha to common target genes is differentially regulated in neuroblastoma: HIF-2alpha promotes an aggressive phenotype. Cancer Cell.

[122] Niedobitek G, Fahraeus R, Herbst H, Latza U, Ferszt A, Klein G (1992). The Epstein-Barr virus encoded membrane protein (LMP) induces phenotypic changes in epithelial cells. Virchows Arch B Cell Pathol Incl Mol Pathol.

[123] Israel BF, Gulley M, Elmore S, Ferrini S, Feng W, Kenney SC (2005). Anti-CD70 antibodies: a potential treatment for EBV + CD70-expressing lymphomas. Mol Cancer Ther.

[124] Kang JS, Bae SY, Kim HR, Kim YS, Kim DJ, Cho BJ (2009). Interleukin-18 increases metastasis and immune escape of stomach cancer via the downregulation of CD70 and maintenance of CD44. Carcinogenesis..

[125] Starzer AM, Berghoff AS (2020). New emerging targets in cancer immunotherapy: CD27 (TNFRSF7). ESMO Open.

[126] Tannir NM, Forero-Torres A, Ramchandren R, Pal SK, Ansell SM, Infante JR (2014). Phase I dose-escalation study of SGN-75 in patients with CD70-positive relapsed/refractory non-Hodgkin lymphoma or metastatic renal cell carcinoma. Investig New Drugs.

[127] Kunle T, Arif O, Walter H, Stadler M, Smith DC, Kluger H (2016). First-in-human multicenter phase I study of BMS-936561 (MDX-1203), an antibody-drug conjugate targeting CD70. Cancer Chemother Pharmacol.

[128] Pal SK, Forero-Torres A, Thompson JA, Morris JC, Chhabra S, Hoimes CJ (2019). A phase 1 trial of SGN-CD70A in patients with CD70-positive, metastatic renal cell carcinoma. Cancer..

[129] Massard C, Soria J-C, Krauss J, Gordon M, Lockhart AC, Rasmussen E (2019). First-in-human study to assess safety, tolerability, pharmacokinetics, and pharmacodynamics of the anti-CD27L antibody-drug conjugate AMG 172 in patients with relapsed/refractory renal cell carcinoma. Cancer Chemother Pharmacol.

[130] Diamantis N, Banerji U (2016). Antibody-drug conjugates—an emerging class of cancer treatment. Br J Cancer.

[131] Nejadmoghaddam M-R, Minai-Tehrani A, Ghahremanzadeh R, Mahmoudi M, Dinarvand R, Zarnani A-H (2019). Antibody-drug conjugates: possibilities and challenges. Avicenna J Med Biotechnol.

[132] Lo Nigro C, Macagno M, Sangiolo D, Bertolaccini L, Aglietta M, Merlano MC. NK-mediated antibody-dependent cell-mediated cytotoxicity in solid tumors: biological evidence and clinical perspectives. Ann Transl Med 2019;7(5):1055. 10.21037/atm.2019.01.42 PMC6462666 31019955

[133] Aftimos P, Rolfo C, Rottey S, Offner F, Bron D, Maerevoet M (2017). Phase I dose-escalation study of the anti-CD70 antibody ARGX-110 in advanced malignancies. Clin Cancer Res.

[134] Awada A, Rolfo CD, Rottey S, Ysebrant de Lendonck L, Schroyens WA, Offner F (2014). A phase I, first-in-human study of ARGX-110, a monoclonal antibody targeting CD70, a receptor involved in immune escape and tumor growth in patients with solid and hematologic malignancies. J Clin Oncol.

[135] Riether C, Pabst T, Höpner S, Bacher U, Hinterbrandner M, Banz Y (2020). Targeting CD70 with cusatuzumab eliminates acute myeloid leukemia stem cells in patients treated with hypomethylating agents. Nat Med.

[136] Noguchi A, Kaneko T, Naitoh K, Saito M, Iwai K, Maekawa R (2014). Impaired and imbalanced cellular immunological status assessed in advanced cancer patients and restoration of the T cell immune status by adoptive T-cell immunotherapy. Int Immunopharmacol.

[137] Fournier C, Martin F, Zitvogel L, Kroemer G, Galluzzi L, Apetoh L (2017). Trial watch: adoptively transferred cells for anticancer immunotherapy. Oncoimmunology..

[138] Donnadieu E, Dupré L, Pinho LG, Cotta-de-Almeida V (2020). Surmounting the obstacles that impede effective CAR T cell trafficking to solid tumors. J Leukoc Biol.

[139] Ochsenbein AF, Riether C, Bacher U, Müller R, Höpner S, Banz Y (2018). Argx-110 targeting CD70, in combination with Azacitidine, shows favorable safety profile and promising anti-leukemia activity in newly diagnosed AML patients in an ongoing phase 1/2 clinical trial. Blood..

[140] Trudel GC, Howes AJ, Jeste N, Tryon JJ, Xiu L, Kane C, Nottage K. CULMINATE: A phase II study of cusatuzumab + azacitidine in patients with newly diagnosed AML, ineligible for intensive chemotherapy. J Clin Oncol. 2020;38:15_suppl, TPS7565-TPS7565.

[141] Gail J. Roboz, Thomas Pabst AA. Safety and efficacy of Cusatuzumab in combination with Venetoclax and Azacitidine (CVA) in patients with previously untreated acute myeloid leukemia (AML) who are not eligible for intensive chemotherapy; an open-label, multicenter, phase 1b study. ASH Annual Meeting Exposition 2021. https://ash.confex.com/ash/2021/webprogram/Paper150371.html

[142] DiNardo CD, Pratz K, Pullarkat V, Jonas BA, Arellano M, Becker PS (2019). Venetoclax combined with decitabine or azacitidine in treatment-naive, elderly patients with acute myeloid leukemia. Blood..

[143] Riether C, Chiorazzo T, Johnson AJ, Drenberg CD, Syed KW, Moshir M (2019). The combination of the BCL-2 antagonist Venetoclax with the CD70-targeting antibody Cusatuzumab synergistically eliminates primary human leukemia stem cells. Blood..

[144] De Meulenaere A, Vermassen T, Creytens D, De Keukeleire S, Delahaye T, Deron P (2021). An open-label, nonrandomized, phase Ib feasibility study of cusatuzumab in patients with nasopharyngeal carcinoma. Clin Transl Sci.

[145] Tu S, Zhou X, Guo Z, Huang R, Yue C, He Y (2019). CD19 and CD70 dual-target chimeric antigen receptor T-cell therapy for the treatment of relapsed and refractory primary central nervous system diffuse large B-cell lymphoma. Front Oncol.

[146] An X, Tiwari AK, Sun Y, Ding P-R, Ashby CR, Chen Z-S (2010). BCR-ABL tyrosine kinase inhibitors in the treatment of Philadelphia chromosome positive chronic myeloid leukemia: a review. Leuk Res.

[147] Lindgren T, Stigbrand T, Riklund K, Johansson L, Eriksson D (2012). Gene expression profiling in MOLT-4 cells during gamma-radiation-induced apoptosis. Tumor Biol.

[148] Pratt D, Pittaluga S, Palisoc M, Fetsch P, Xi L, Raffeld M (2017). Expression of CD70 (CD27L) is associated with Epithelioid and Sarcomatous features in IDH-wild-type Glioblastoma. J Neuropathol Exp Neurol.

[149] De Keersmaecker B, Claerhout S, Carrasco J, Bar I, Corthals J, Wilgenhof S (2020). TriMix and tumor antigen mRNA electroporated dendritic cell vaccination plus ipilimumab: link between T-cell activation and clinical responses in advanced melanoma. J Immunother Cancer.

[150] Ansell SM, Flinn I, Taylor MH, Sikic BI, Brody J, Nemunaitis J (2020). Safety and activity of varlilumab, a novel and first-in-class agonist anti-CD27 antibody, for hematologic malignancies. Blood Adv.

